# Pharmacological methods for reducing coughing on emergence from elective surgery after general anesthesia with endotracheal intubation: protocol for a systematic review of common medications and network meta-analysis

**DOI:** 10.1186/s13643-019-0947-2

**Published:** 2019-01-24

**Authors:** Alan Tung, Nicholas A. Fergusson, Nicole Ng, Vivien Hu, Colin Dormuth, Donald G. E. Griesdale

**Affiliations:** 1 Department of Anesthesiology, Pharmacology and Therapeutics, University of British Columbia, Vancouver, Canada; 2 Faculty of Medicine, University of British Columbia, Vancouver, Canada

**Keywords:** Cough, General anesthesia, Extubation, Emergence, Network meta-analysis, Systematic review

## Abstract

**Background:**

Emergence coughing and bucking, secondary to endotracheal tube stimulation of the tracheal mucosa, frequently occurs after the general anesthetic recedes. Besides general unpleasantness, coughing has important physiological sequelae that may be detrimental to the postoperative patient. Multiple pharmacological strategies have been published, but prior systematic reviews on this topic have neither been comprehensive enough in their literature or medication search, nor provided us the answer regarding what the best pharmacological method is to prevent or minimize peri-extubation coughing. Our systematic review and network meta-analysis’ primary objective is to determine the relative efficacies of different pharmacological methods on decreasing coughing (none to mild compared to moderate to severe, as defined by the modified Minogue scale) during emergence after a general anesthetic with endotracheal intubation in adult elective surgeries. Medications of interest are lidocaine or lignocaine (intravenous (IV), intracuff alkalinized, intracuff non-alkalinized, topical, endotracheal application), dexmedetomidine IV, remifentanil IV, and fentanyl IV. These medications were selected based on a preliminary review of the literature.

**Methods:**

Using a predefined search strategy, we will search MEDLINE, Cochrane Central Register of Controlled Trials, Embase, Cochrane Database of Systematic Reviews, ACP Journal Club, Database of Abstracts of Reviews of Effects, and the Cochrane Methodology Register, with no date or language restrictions. Gray literature search will encompass conference abstracts, Web of Science, and references from publications selected for full-text review. Two reviewers will independently screen the retrieved literature using predetermined inclusion criteria, process publications selected for full-text review, extract data from publications chosen for study inclusion, and evaluate for bias using the Cochrane risk of bias assessment. Risk ratios and 95% confidence intervals will be calculated for each study, and a surface under the cumulative ranking curve will determine the relative rank of each intervention in its ability to prevent coughing on emergence.

**Discussion:**

The proposed systematic review and network meta-analysis will not only provide a more thorough review of common medications used to decrease emergence coughing, but also inform clinicians which of these pharmacological strategies is the best approach.

**Systematic review registration:**

PROSPERO CRD42018102870

## Background

Coughing and bucking while intubated on emergence from general anesthesia unfortunately occurs in approximately 40% of patients [1, 2]. Coughing ensues as the effects of anesthesia recede and permit greater peripheral and central nervous system perception of the endotracheal tube stimulating the trachea [3]. In addition to being uncomfortable, coughing has important physiological consequences: increased intrathoracic pressure, decreased venous return to the right atrium, increased intra-abdominal pressures, decreased functional residual capacity, and increased blood pressure [4].

While most patients do not experience significant sequelae, minimizing coughing on emergence should be emphasized in certain situations. Patient populations with poor respiratory function, such as obese patients with decreased functional reserve capacity, may develop hypoxemia secondary to post-tussive atelectasis. Particular surgeries should also have a “smooth emergence” with minimal coughing to avoid complications: thyroidectomy (surgical bleeding) [5], laparotomy or hernia repairs (wound dehiscence) [6], carotid endarterectomy (neck hematoma), and intracranial surgery (intracerebral hemorrhage) [7].

Pharmacological methods to decrease coughing peri-extubation are well published in the literature [4, 8]. However, most of these studies utilize small study population and administer the medication at various doses and routes. Few studies did head-to-head comparisons between different pharmacological methods. Prior meta-analysis publications have predominantly examined the use of intracuff lidocaine to manage emergence coughing [9, 10]. Jubb and Ford performed a systematic review examining lidocaine, remifentanil, alfentanil, verapamil, and beta-blockers [8]. However, they did not include studies which examined dexmedetomidine. Furthermore, their search was limited to a single database, thereby subjecting the results to selection bias. Given the multitude of publications on various medical strategies to decrease peri-extubation coughing, the increased interest in dexmedetomidine, and the lack of clarity on what is the best evidence-based pharmacological strategy, a more thorough review of the published data on minimizing emergence coughing is warranted.

With a systematic review, we aim to gather published and gray literature available online to comprehensively review common medications tested in controlled trials to reduce coughing on emergence. Medications of interest include lidocaine (intravenous (IV), intracuff (alkalinized and non-alkalinized), endotracheal, and topical applications), dexmedetomidine IV, remifentanil IV, and fentanyl IV. These medications were identified as the most common, published strategies during a preliminary screen of the literature. If feasible, a network meta-analysis will help us compare the relative efficacy of each pharmacological method, a question that otherwise would have been difficult to answer given the paucity of trials comparing different medications and routes in head-to-head trials.

## Methods

This protocol was developed following the Preferred Reporting Items for Systematic Review Protocols (PRISMA-P) Statement [11] and is registered with the International Prospective Register of Systematic Reviews (PROSPERO) database (CRD42018102870) [12]. Any modifications to the protocols will be described in the publication of the final report. The PRISMA extension for the network meta-analysis will also be utilized in preparation for the final report to ensure all aspects of the methods and findings are reported [13].

### Objectives

The primary goal of this systematic review and network meta-analysis is to determine the relative efficacies of different pharmacological methods in decreasing the incidence of moderate to severe coughing on extubation in adult elective surgeries conducted under general anesthesia with endotracheal intubation. Coughing severity will be based on the modified Minogue scale (Table 1) [14].

**Table 1:** Original and modified Minogue scales and outcomes categorization for the primary outcome analysis

Outcomes categorization	Grade and severity description	Modified Minogue scale To be used for our analysis	Original Minogue scale 3-point Likert scale
None to mild	Grade 1 (none)	No coughing or muscular stiffness	
	Grade 2 (mild)	Coughing once or twice, or transient cough response to removal of tracheal tube that resolved with extubation	Single cough
Moderate to severe	Grade 3 (moderate)	≤ 3 coughs lasting 1~2 s, or total duration of coughing last ≤ 5 s	More than one episode of non-sustained coughing lasting ≤ 5 s
	Grade 4 (severe)	≥4 coughs with each lasting > 2 s, total duration of coughing last > 5 s	Sustained coughing of more than 5 s duration

Secondary objectives include comparisons of the frequencies of zero, mild, moderate, and severe coughing on emergence, and the extubation times (defined as the start of emergence to extubation) among the different pharmacological strategies. Other secondary objectives include the effects of total intravenous (TIVA) versus volatile maintenance anesthetic, and high versus low study medication regimens, on the frequency of moderate and severe peri-extubation coughing. Designation of high or low dose regimens for each study medication will be determined by identifying the total median dose of all selected studies and subsequently splitting the results based on whether the dose used was below or above the median dose.

Lastly, our tertiary objectives include determining the hemodynamic sequelae from the pharmacological methods, and complications or adverse events associated with the use of the medications of interest. Hemodynamic data include systolic blood pressure, diastolic blood pressure, mean arterial pressure, heart rate, respiratory rate, and oxygen saturation. Complications and adverse events may consist of events, other than hemodynamic events, that result in morbidity, mortality, or medical or surgical intervention.

### Data sources and search for studies

We will use the OVID interface to access the main biomedical-related databases for our search for relevant publications using predefined search strategies: MEDLINE, CENTRAL, EMBASE, Cochrane Database of Systematic Reviews, ACP Journal Club, Database of Abstracts of Reviews of Effects, and Cochrane Methodology Register (see Appendix, *Search strategy* below). There will be no date cut-off, with all database searches commencing from the database’s inception to present. The key words will be related to cough, anesthesia, a trial of some sort, and the medications of interest (lidocaine or lignocaine, remifentanil, dexmedetomidine, and fentanyl). The searches will be combined with the AND Boolean logic operator. The search strategy will be reviewed by the authors. No search filters will be applied. The proposed list of search terms is listed in the Appendix section.

As part of the gray literature search, we will search the Web of Science Core Collection (inception to present) using the topic search terms related to cough, extubation, and anesthesia (see Appendix). We will also obtain conference abstracts published online from the American Society of Anesthesiologists (2000~2017), the Canadian Anesthesiology Society (2001~2017), and the European Society of Anesthesiologists (2004~2017). All publications selected for full-text review will have their references searched for additional potentially relevant publications.

The literature search strategy using MEDLINE will be based on a combination of MeSH terms, text word, and publication types. For other databases (CENTRAL, EMBASE, Cochrane Database of Systematic Reviews, ACP Journal Club, Database of Abstracts of Reviews of Effects, and Cochrane Methodology Register), a combination of text word and all fields will be utilized to ensure an encompassing literature search strategy.

### Study inclusion and exclusion criteria

Inclusion and exclusion criteria have been developed in the PICOS (population, intervention, comparators, outcomes, study design) format.

#### Population

The participants will be adults (18 years or older) who had elective surgery under general anesthesia with an endotracheal intubation. Exclusion criteria include participants who undergo emergency surgery or are pregnant. Animal studies are also excluded.

#### Intervention

The intervention is a pharmacological method (medication, dose, and route) that was administered pre-intubation, intra-operatively, pre-extubation, or during extubation with the intent of smoothing emergence or decreasing coughing on extubation. Medications of focus are lidocaine, remifentanil, dexmedetomidine, and fentanyl.

#### Comparator

The study will compare the intervention to either placebo or a medication of interest.

#### Outcomes

The studies to be included in the systematic review will have collected and presented data on the incidence and/or severity of coughing on extubation during emergence. Emergence is defined as the time period between the complete discontinuation of the main maintenance anesthetic to 5 min post-extubation. Cough grading will be based on the modified 4-point Minogue scale (Table 1). Grade 1 equates to no cough; grade 2 (mild) represents coughing once or twice; grade 3 (moderate) means fewer than 4 non-sustained coughs lasting 1~2 s each or overall coughing lasting less than 5 s; and grade 4 (severe) is at least 4 coughs lasting at least 2 s, or overall coughing duration being more than 5 s [15]. For the purpose of the primary outcome, all patients with grades 1 and 2 coughing will be categorized as “none to mild”, while other patients with grades 3 and 4 will be categorized as “moderate to severe”. For studies that only report the incidence of coughing, then all patients that had peri-extubation coughing will be placed in the “moderate to severe” outcomes category, while the rest (who did not cough) will be in the “none to mild” section. These studies will only be used for the primary outcome and not the secondary objective examining the frequencies of the different grades.

If the study utilizes another coughing scale, then attempts would be made to fit the results into the modified Minogue scale based on the scale’s description of the coughing grades. If the description is not available or vague, or if the study’s coughing grades are not possible to match to the modified Minogue scale, then the study’s results will be treated as if the study only reported on incidence.

#### Study design/characteristics

Studies collected for the systematic review will be single-blinded or double-blinded, randomized controlled trials. The publications can be published in any language and from any date. The studies need to report peri-extubation coughing as incidence of severity frequency on a 3-point, 4-point, or 5-point scale. Excluded studies will either have duplicate reporting of patient cohorts, or have appropriate data that cannot be extracted, calculated from the published data, or obtained from the original authors. Studies that used supraglottic airway devices (i.e. laryngeal mask airways) are also excluded from this study.

### Screening and data extraction

The screening process starts with obtaining the titles and abstracts of all publications identified by the database searches using the predetermined search strategy noted above. Two reviewers will independently screen the titles and abstracts for potential relevance. If the trial or publication may fulfill our eligibility criteria, then the full text will be obtained and independently reviewed by two reviewers. Any discrepancies or disputes on eligibility will be resolved by discussion between the two reviewers and/or a third reviewer. Reasons for inclusion or exclusion of a publication or trial will be provided for all studies that have undergone a full-text review. A PRISMA flow diagram will be created to document the study selection process in the final publication.

A prespecified data extraction form designed by the primary author using Microsoft Word (Microsoft Corporation, Seattle, Washington, USA) will be used to collect data. The form will be piloted on the first five selected studies, and form will be modified as needed. Data will be extracted from the publication, supplementary material, and/or information provided by the study author if deemed necessary. All studies selected for full-text review will be assessed and their data extracted by two independent reviewers. Any discrepancy or disagreement will be resolved by discussion and/or a third reviewer.

Utilizing the PICOS framework, we will extract the following data: publication characteristics (title, authors, publication reference); study details (setting, time period of study, design, primary objectives, randomization); population characteristics (age, sex, height, weight, BMI, smoker status, surgery and/or anesthesia durations, type of surgery, ASA physical status classes, inclusion/exclusion criteria, anesthetic maintenance type (total intravenous anesthetic versus inhalational volatile)); intervention (medication, dose, route, timing of administration (pre-intubation, intraoperatively, emergence, during extubation), number of patients who had received the intervention); comparator (medication, dose, route, timing of administration, number of patients who had received the comparator method); primary outcomes (definition of cough, frequency of cough, blinding status); secondary outcomes (severity grading scale of coughing; blinding status of observer; any reporting of statistically significant hemodynamic changes (heart rate, systolic blood pressure, diastolic blood pressure, respiratory rate, oxygen saturation)) relative to comparator and to baseline; time to emergence; and adverse events or complications resulting in morbidity, mortality, and/or medical or surgical intervention.

### Risk of bias assessment

Two reviewers, for each study selected for full-text review, will use the Cochrane Collaboration’s tool for assessing risk of bias in randomized trials [13]. Reviews will be compared for consistency, and any disagreements will be resolved by discussion between the two reviewers or after consultation with a third reviewer. The primary domains assessed by the Cochrane risk of bias tool will be the selection bias (random sequence generation), allocation sequence concealment, performance bias (blinding of participants and researchers), detection bias (blinding of outcome assessment), attrition bias (incomplete outcome data), reporting bias (selective reporting), and other biases if found.

Furthermore, we will visually assess for publication bias using a network funnel plot.

### Approach to evidence synthesis

Study characteristics (i.e. age, sex, height, weight, BMI, smoker status, surgery duration, anesthesia duration, type of surgery, ASA physical status classes, and anesthetic maintenance type) and risk of bias will be examined and summarized. We will calculate the risk ratio (RR) and the 95% confidence interval for each study. The magnitude of statistical heterogeneity within each pair-wise comparison will be assessed using the *I*^2^ measure. Values of *I*^2^ greater than 75% will be considered as indicative of a high degree of heterogeneity. Study design, patient characteristics, and interventions will also be considered when determining if meta-analyses will be conducted. We will display the treatment effects for pair-wise comparisons in the network using a Forest Plot. Furthermore, a surface under the cumulative ranking curve (SUCRA) analysis will be conducted to determine the relative rank of each intervention in the ability to prevent cough on emergence.

We will test the consistency assumption using both overall and local methods. Overall inconsistency will be assessed using the STATA command *network meta inconsistency.* There will be an omnibus test of overall consistency using a Wald test. We will then test for local inconsistency using the STATA command *network sidesplit all*. A consistency model will be used only when there is no inconsistency on both overall and local tests. Publication bias will be visually assed using a network funnel plot. Lastly, we will perform a subgroup analysis of studies that are either high risk of bias or conference abstracts, which often contain limited information, to evaluate the risk of bias of including such studies.

## Discussion

Emergence coughing has important physiological and serious health consequences, ranging from post-tussive atelectasis and desaturation to surgical failure such as wound dehiscence in a ventral hernia repair with a component separation technique. Numerous pharmacological methods to decrease peri-extubation coughing have been published. However, few studies have compared these medications in head-to-head trials. Furthermore, previous published systematic reviews on this topic have not been comprehensive enough in scope of both the medications and available literature. Our study is designed to address both issues. Lidocaine, remifentanil, dexmedetomidine, and fentanyl were selected for our study for two key reasons: (1) they are the most common medications used, based on our preliminary scan of the literature, and (2) these drugs are readily available in many hospitals and operating rooms, as substantiated by the international origins of the studies selected for full-text review. While pairwise meta-analyses may have been the traditional way to compare the effectiveness of interventions, a network meta-analysis can synthesize data, from both direct head-to-head comparison trials and indirect evidence from multiple comparators, into a cohesive analysis. This analysis will provide a superior, more complete answer as to what pharmacological strategy is the best way to decrease moderate to severe coughing on emergence from general anesthesia with endotracheal intubation in adult elective surgery, with the goal of avoiding complications from coughing.

Extubation times and hemodynamic profiles are also important for clinicians when selecting an agent to decrease coughing. Prolonged extubation times may slow the turnover of an operating room and subsequently may delay cases, cancel surgeries, and impact patient care. Remifentanil and dexmedetomidine are known to cause bradycardia and hypotension, which may be undesirable in patients with hemodynamic instability. Therefore, gathering and summarizing this information will help clinicians assess the risks and benefits of using these medications.

We aim to submit our results to a peer-reviewed publication and present our findings at national and international meetings and conferences.

## Appendix

Search strategy—sample for one database

MEDLINE

1. Cough* [Text Word]
2. Buck* [Text Word]
3. Cough [Subject Headings]
4. Coughs [Subject Headings]
5. 1 or 2 or 3 or 4
6. Trial* [Text Word]
7. Random* [Text Word]
8. Clinical trial [Subject Headings]
9. Clinical trial as topic [Subject Headings]
10. Controlled clinical trial [Subject Headings]
11. Controlled clinical trials as topic [Subject Headings]
12. Randomized controlled trial [Subject Headings]
13. Randomized controlled trials as topic [Subject Headings]
14. Multicentre trial [Subject Headings]
15. Multicentre trials [Subject Headings]
16. Multicentre studies as topic [Subject Headings]
17. Multicenter trial [Subject Headings]
18. Multicenter trials [Subject Headings]
19. Multicenter studies [Subject Headings]
20. Allocation, random [Subject Headings]
21. Trials, randomized clinical [Subject Headings]
22. Double blind method [Subject Headings]
23. Double blind methods [Subject Headings]
24. Double blind study [Subject Headings]
25. Double blind studies [Subject Headings]
26. Method, single blind [Subject Headings]
27. Methods, single blind [Subject Headings]
28. Single blind study [Subject Headings]
29. Single blind studies [Subject Headings]
30. Prospective studies [Subject Headings]
31. Prospective study [Subject Headings]
32. Clinical trial [Publication Type]
33. Controlled clinical trial [Publication Type]
34. Randomized controlled trial [Publication Type]
35. Multicenter study [Publication Type]
36. Multicentre study [Publication Type]
37. 6 or 7 or 8 or 9 or 10 or 11 or 12 or 13 or 14 or 15 or 16 or 17 or 18 or 19 or 20 or 21 or 22 or 23 or 24 or 25 or 26 or 27 or 28 or 29 or 30 or 31 or 32 or 33 or 34 or 35 or 36
38. Anesthesia [Text Word]
39. Anaesthesia [Text Word]
40. Anesthetic [Text Word]
41. Anaesthetic [Text Word]
42. Anesthesia [Subject Headings]
43. Anesthesia, general [Subject Headings]
44. Anesthesia recovery period [Subject Headings]
45. Anesthesia recovery periods [Subject Headings]
46. 38 or 39 or 40 or 41 or 42 or 43 or 44 or 45
47. Extubat* [Text Word]
48. Emergence [Text Word]
49. Airway extubation [Subject Headings]
50. Airway extubations [Subject Headings]
51. Endotracheal extubation [Subject Headings]
52. Endotracheal extubations [Subject Headings]
53. Intratracheal extubation [Subject Headings]
54. Intratracheal extubations [Subject Headings]
55. Tracheal extubation [Subject Headings]
56. Tracheal extubations [Subject Headings]
57. 47 or 48 or 49 or 50 or 51 or 52 or 53 or 54 or 55 or 56
58. Lidocaine [Text Word]
59. Lignocaine [Text Word]
60. Dexmedetomidine [Text Word]
61. Remifentanil [Text Word]
62. Fentanyl [Text Word]
63. 58 or 59 or 60 or 61 or 62
64. 5 and 37 and 46 and 57 and 63

## Abbreviations

IV: Intravenous
PICOS: Population, intervention, comparator, outcome, study
PRISMA: Preferred Reporting Items for Systematic Review Protocols
PROSPERO: International Prospective Register of Systematic Review
RR: Risk ratio
SUCRA: Surface under the cumulative ranking curve
